# Chinese medicine and biomodulation in cancer patients—Part two

**Published:** 2008-04

**Authors:** S.M. Sagar, R.K. Wong

**Affiliations:** Departments of Oncology and Medicine, McMaster University; Juravinski Cancer Program, Hamilton Health Sciences Corporation; and The Brain–Body Institute, St. Joseph’s Healthcare System, Hamilton, Ontario, Canada

**Keywords:** Chinese medicine, herbs, acupuncture, supportive care, immunity, research

URL: http://www.current-oncology.com/index.php/oncology/article/view/238/214

## E-JOURNAL LINKED ABSTRACT

Traditional Chinese Medicine (tcm) is a whole system containing therapeutic interventions that individually induce biomodulation at the physiologic, chemical, and molecular levels. The theory of tcm proposes a synergy between specific interventions selected as part of a care plan based on tcm diagnostic theory. Combining tcm with the modern practice of oncology seems, in conjunction with biomedical interventions (surgery, radiotherapy, chemotherapy, and pharmaceuticals), to have potential advantages through the synergy of biomodulation. Biomodulation approaches are broadly categorized as modification of tumour response and reduction of adverse effects; modulation of immunity; prevention of cancer progression; and enhancement of symptom control. Although the database of preclinical studies is rapidly expanding, good-quality clinical trials are notably scarce.

Laboratory studies suggest that some herbs increase the effectiveness of conventional chemotherapy without increasing toxicity. A healthy immune system is necessary for control of malignant disease, and the immune suppression associated with cancer contributes to its progression. Many Chinese herbs contain glycoproteins and polysaccharides (among them, constituents of *Coriolus versicolor, Ganoderma lucidum, Grifola frondosa, Astragalus membranaceus, Panax ginseng,* and various other medicinal mushrooms) that can modulate metastatic potential and the innate immune system. Phytochemicals such as specific polysaccharides have been shown to boost the innate immune system, especially through interaction with Toll-like receptors in mucosa-associated lymphoid tissue. This intervention can potentially improve the effectiveness of new anticancer vaccines. An increase in virus-associated cancers presents a major public health problem that requires novel therapeutic strategies. A number of herbal therapies have both antiviral activity and the ability to promote immunity, possibly inhibiting the initiation and promotion of virus-associated cancers.

The mechanisms learned from basic science should be applied to clinical trials both of specific interventions and of whole-system care plans that safely combine the tcm approach with the conventional biomedical model. In Western medicine, the combination of tcm herbs with drug therapies is controversial, given lack of knowledge concerning whether a drug is favourably enhanced or whether adverse effects occur. Using initial data from the preclinical studies, future clinical research needs to evaluate the combinations, some of which are showing favourable synergy.

## FEATURED READING TOOLS

For in-depth information, readers may want to visit these sites: Society for Integrative Oncology (SIO)

Shanghai International Symposium: Integrative Oncology Theory, Research, and Practice April 25–26, 2008, Shanghai, China (www.siosh.org/en/index.asp) and Society for Integrative Oncology (www.integrativeonc.org/)

